# Factors influencing the satisfaction of community senior care services in China: an analysis based on an extended Anderson model

**DOI:** 10.3389/fpubh.2023.1138711

**Published:** 2023-06-23

**Authors:** Wei Ma, Junfeng Wang, Lihong Liu, Han Zhang

**Affiliations:** ^1^School of Public Policy and Administration, Xi’an Jiaotong University, Xi’an, Shaanxi, China; ^2^School of Public Policy and Management, University of Illinois Springfield, Springfield, IL, United States

**Keywords:** extended Anderson behavior model, utilization of senior care services, satisfaction, fairness perception extended Anderson behavior model, fairness perception

## Abstract

Community-based senior care, as a convenient and promising care model, has gradually been accepted by the public. However, community services developed to facilitate older adults often fail to achieve the expected effect. With the fast-growing aging population in China, the problems of underutilization and low service satisfaction of senior care facilities need to be resolved urgently. In this study, we further developed an extended Anderson behavior model by incorporating social psychological factors, and the vertical and horizontal fairness perceptions. In addition, a binary logistic regression model was used to analyze factors affecting the satisfaction of older adults in life care services, health care services, and mental and spiritual comfort services. The study used data from a survey of 322 urban area seniors in Shaanxi Province. The results showed that factors influencing older adults’ satisfaction of different service categories are different. Moreover, with the addition of the social psychological factors, we observed that the vertical fairness perception of the survey respondents affected their satisfaction of senior care services significantly more than the horizontal fairness perception.

## 1. Introduction

Population aging has been a crucial policy issue globally. In the past two decades, the fertility rate in China has continuously reduced, and the aging population structure has become increasingly serious. The Fifth Chinese Population Census in 2000 revealed that China had entered an aging society. After 20 years, according to the latest Seventh Chinese Census in 2021, the proportion of population aged ≥60 years accounts for 18.7% of the total (1). Moreover, researchers have forecasted that in the following decades, the population aging rate in China will continue to accelerate and maintain a high-speed growth after reaching its peak around 2060, which could be termed as a “plateau” trend (2).

With a low fertility rate, traditional family care in China has become challenging. Family care is insufficient to meet the care needs of older adults. In 2015, the demand for senior care services in urban and rural areas reached 15.3%, which considerably increased compared with 12.37% in 2010 and 6.6% in 2000 (3). Therefore, meeting the senior care needs has become an urgent problem for the Chinese government. Community care service, as a more “win–win” option, has become the most widely used senior care model in China. Combining the advantages of traditional family care and institutional care, the community can be seen as a platform for connecting professional institutions and nursing staff with older adults at home. This means older adults can receive medical and non-medical services and solve their daily living problems in the community or even at home.

Pardasani and Thompson (4) studied six types of community care services, namely community center, wellness center, lifelong learning, continuum of care, entrepreneurial center, and the café program. In China, community care services mainly comprise the first three types (5). With the continuous expansion of the older population, the demand for senior care service is gradually showing a diversified and differentiated trend, thereby pushing communities to upgrade their service contents and quality. However, numerous studies have revealed the persistence of problems of underutilization and low service satisfaction in senior care service [(6, 7); Hu et al., 2019].

First, from the supply and demand perspective, the choice of current senior care service is limited (8). By contrast, because of factors such as regional culture, income level, age, and gender, the service demand among older adults is quite different. Therefore, a gap exists between the supply of senior care services and the diversified and differentiated care service needs of older adults (9). The current senior care services often cannot accurately assess “need,” “unneeded,” and “want” from the older adults, thus resulting in an imbalance in the supply and demand structure of senior care service (10). Second, from the perspective of characteristics of older adults, differences in factors such as age, gender, marital status, income level, and self-care status (11) have an impact on the satisfaction with senior care services. Similarly, the attitude and acceptance toward social senior care services among older adults significantly affect the utilization of these services. A study revealed that people less influenced by the filial piety culture were more likely to use the senior care services to a great extent (12). Third, social psychological factors such as social trust, social capital, social integration, social equality, and social participation significantly affect the satisfaction with senior community care services (13). Specifically, older adults experience psychosocial problems such as social isolation, identity threat, and lack of social support, all of which affect the senior care intention (14).

The current research on the satisfaction with utilization of senior care service focuses more on personal characteristics, service quality, and affordability but relatively less on social psychological factors. This paper constructs a model by adding another dimension about social psychosocial factors in the traditional Anderson model. Using binary logistic regression analysis, this study explored the main factors that affect the satisfaction with community senior care services. The findings hopefully provide some effective suggestions for improving the community senior care service utilization rate and realize the Chinese social vision of “the older will be looked after properly.”

## 2. Methodology

### 2.1. Analysis framework

The Anderson behavior model was used to study the conditions facilitating or impeding the utilization of personal medical services (15). Over the years, the model has undergone continuous improvement. It has now been extended to analyze the actual or expected use of various services by different people groups (16). The traditional framework focused on a series of predisposing, enabling, and need factors influencing service utilization. Over the years, scholars have modified the model for answering different research questions. By combining the Anderson behavior model with the P-E fit theory, Wu found that interpersonal, spatial, and information linkage would have a great impact on facilitating service utilization in the process of older adults adapting to a new environment (17). According to the P-E fit theory, older adults using community care services is more of a process of their re-adaptation to the social environment, which includes two aspects, adaptation to the organization and interaction among people. As the linkages with the community are becoming tighter, older adults would more likely to become comfortable with their new identities and be able to better utilize community resources in meeting their needs. Yu et al. (18) reported that the Anderson behavior model has been studied mostly from the demand perspective and not from the supply perspective. According to the previous research (Yu et al., 2021), the Anderson behavior model has been studied mostly from the demand perspective and not from the supply perspective. The quality, price, and accessibility of the service would significantly impact the demand from elders. These findings are consistent with those of one study reporting that an increment in community senior care services would in turn increase the demand for these services (19).

Bradley showed that although the traditional Anderson behavior model referred to the concept of “faith” in predisposing factors, it mainly discussed about factors such as older adults’ view on diseases and health services, while focusing little on social psychological factors (20). Shi also demonstrated that the psychological perception of older adults would influence the supply–demand satisfaction. On the one hand, older adults’ psychological perception about basic living needs, living environment, personal traits, and livability for the aged would directly influence their satisfaction as clients (21). On the other hand, in the process of using community-based senior care service, perceptions of inequality, independence of consciousness, social trust, tradition, and other cultural values would affect older adults’ attitude and action about accepting social help (13, 22, 23). Moreover, based on the results of Bradley’s research, Zeng extended the model with social psychological factors and found that intergenerational ties, unmet needs for Long-Term Care (LTC), and self-image evaluation would influence LTC needs (24). Therefore, fairness perception was examined as a psychological factor in our model.

**Figure 1:**
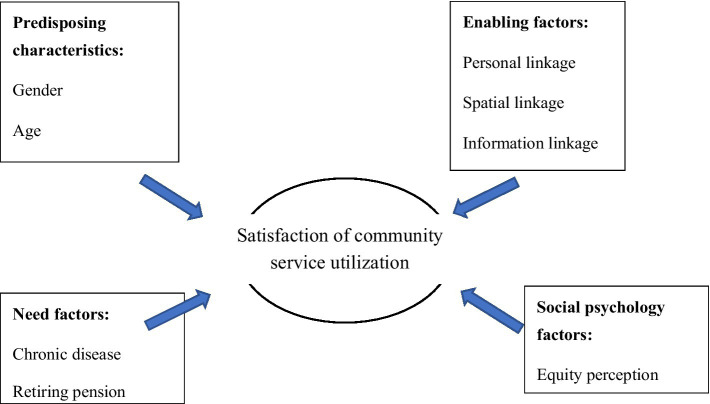
Analytical framework of community service utilization satisfaction.

Studies have often adopted equity theory to analyze the reasons for social help from social psychology perspectives (25). In most studies about fairness, scholars have focused on the inequity status in the utilization process, such as the unequal utilization status because of race, gender, income, etc. (26–28). Fewer studies have examined whether differences exist in older adults’ subjective perception of fairness, which thus affects their community service utilization satisfaction. Wang et al. found that with age, people are more inclined to exhibit interpersonal tendencies in social interactions, that is, when interacting or comparing with other old people, they become more tolerant of unfair treatment and become less competitive (29). Therefore, this study incorporated the perception of fairness as a social psychological factor into the research framework model. More or less inequity in service supply will be observed in the actual service utilization process. This study divided old people’s resonance of unequal treatments into two dimensions: the perception of vertical and horizontal injustice. We propose that vertical injustice more significantly influences service utilization than horizontal injustice. To summarize, an extended Anderson behavior model can be established for the satisfaction about community healthcare service utilization among older adults, as shown in Figure 1.

This study extends the traditional Anderson behavior model from three dimensions to four dimensions by adding social psychological factors as a dimension. Based on this model, we examined how fairness perception has an impact on older adults’ satisfaction of community-based senior service. Moreover, compared with a previous study, this study discusses not only the utilization of medical community healthcare services but also the non-medical community healthcare service with life care service satisfaction and spiritual comfort service satisfaction. By comparing the differences between their contributing factors, we can make more targeted recommendations.

### 2.2. Data and sample

Economic development in Shaanxi Province, located in the west of China, had been limited by its location and fast-growing aging population. The policy support from the central government and new immigrants have recently ensured some promising development in this province. Selecting Shaanxi as the study area expanded our survey scope to reach different senior groups, as Shaanxi is a province of diversity and possibility owing to its unique geographical environment and historical and cultural background. The sample was drawn from 12 urban communities in three cities of this province. In total, 340 urban community residents aged ≥60 years responded to the survey. Later, 18 responses were deemed invalid, resulting in the inclusion of 322 survey responses in this study.

### 2.3. Dependent variables

Our dependent variable is satisfaction with the community-based senior care service. This study analyzed the differences in factors influencing various community service types. Considering both physical and psychological service demands, we categorized community-based service for senior into three types: life care service, health care service, and spiritual and mental care service. The questions such as “how satisfied are you with life care services/healthcare services/mental and spiritual service provided by the community?” were asked to assess the outcome. We here recoded the variables so that “very satisfied” and “satisfied” were uniformly categorized as “satisfied,” and “neutral,” “unsatisfied,” and “strongly unsatisfied” were categorized as “not satisfied.”

### 2.4. Independent variables

#### 2.4.1. Predisposing variables

Predisposing factors were typically associated with demographic, social structure, and health beliefs. Age, gender, education, and marital status were included to measure biological and social imperatives.

#### 2.4.2. Enabling variables

Enabling factors refer to the accessibility of service resources among older adults. From the Wu’s perspective, when old people attempt to use community care services, they are re-adapting to the environment after their retirement. In this process, when older adults reach their optimal state with the environment, they become more willing to use senior care services. Therefore, this study primarily focused on personnel, spatial, and information linkage factors to indicate the basic conditions of older adults and the environment whether it would enable or impede their senior care service utilization. First, closeness with others was the crucial predictor for measuring personal linkage. Personal linkage was measured through the question “how frequently do you see your family and interact with neighborhoods and friends.” Spatial linkage mainly pointed to the accessibility of community-based aging service and was measured through the question “the convenience you went to senior service organization(s) such as the Community Senior Service Center, Community Health Care Center and Community Recreation Center.” Lastly, information linkage, a crucial method for older adults to adapt the environment, was measured on the basis of the index of seniors’ proficiency in using smartphones. The answers for these three questions included “not close/inconvenient/strange (1),” “close/convenient/proficient (2),” and “very close/convenient/proficient (3).”

#### 2.4.3. Need variables

Need factors included those caused by the physical function or financial limitations and those perceived service demands. Chronic diseases, retiring pension, and labor income were selected as the indices for use as need variables. We enquired old people whether they have any chronic disease, retiring pension, and labor income. The answers were “yes (1)” and “no (0).”

#### 2.4.4. Social psychological variables

As illustrated in our analytical model (Figure 1), fairness perceptions, including vertical and horizontal fairness perceptions, were introduced in the framework. Vertical fairness perception was measured by comparing whether the services they received matched with their previous contributions to the society, and horizontal fairness perception was measured by comparing whether other old people received the same treatment of senior care services. They were measured on the basis of questions such as “Do you agree that the services you received are equal to those other old people” and “Do you agree that the services you received can match with your contributions to the society.” The answers included “disagree (1),” “agree (2),” and “strongly agree (3).”

The measurements of all variables are shown in Table 1.

**Table 1:** Measurements of variables.

Variable type	Variable name	Variable label	Measurement
Dependent variable			
Service utilization satisfaction	Life care service utilization satisfaction	Overall satisfaction with community-based senior life care service utilization	1 = “not satisfied”；2 = “satisfied”
	Healthcare service utilization satisfaction	Overall satisfaction with community-based senior healthcare service utilization	1 = “not satisfied”；2 = “satisfied”
	Mental and spiritual service utilization satisfaction	Overall satisfaction with community-based senior spiritual and mental service utilization	1 = “not satisfied”；2 = “satisfied”
Independent variable			
Predisposing factors	Age	Age	Age ≥ 60
	Gender	Gender	0 = female；1 = male
	Education	Education	1 = primary school and below, 2 = middle school, 3 = high school and above
	Marital status	Marital status	0 = without spouse (single, divorced or widowed),1 = married
Enabling factors	Spatial linkage	Convenience to community older service center	1 = “inconvenient”；2 = “convenient”；3 = “Very convenient”
		Convenience to community healthcare center	1 = “inconvenient”；2 = “convenient”；3 = “Very convenient”
		Convenience to community recreation center	1 = “inconvenient”；2 = “convenient”；3 = “Very convenient”
	Personal linkage	Closeness to family & friends	1 = “not close”；2 = “close”；3 = “very close”
	Information linkage	Proficiency in using smartphones	1 = “strange”; 2 = “proficient”；3 = “very proficient”
Need factors	Health status	Whether the respondent has any chronic diseases	0 = “No”; 1 = “Yes”
	Retiring pension	Whether the respondent has retiring pension	0 = “No”; 1 = “Yes”
	Labor income	Whether the respondent has labor income	0 = “No”; 1 = “Yes”
Social psychological factors	Fairness perception	Compared with other senior citizens, the fairness about the equal access to all senior care services	1 = “disagree”; 2 = “agree”；3 = “Very agree”
		The received services whether reflect the respondent’s social contribution before retirement	1 = “disagree”; 2 = “agree”；3 = “Very agree”

## 3. Results

### 3.1. Descriptive statistics

The characteristics of predisposing, enabling, and need factors are presented in Table 2. From the perspective of predisposing characteristics, 194 (or 60.2%) respondents were women and 128 (or 39.8%) were men. Age of the majority (87.88%) of the respondents ranged from 60 to 80 years and their physical and cognitive skills functioned relatively well. This group of older adults would be more likely to know and utilize senior care services. In total, 89 (or 27.64%) respondents received elementary school and below education, and 41.3% of the respondents received high school and above education.

**Table 2:** Descriptive statistics about predisposing, enabling, and need factors (*N* = 322).

	Variable categories	*N* (%)		Variable categories	*N* (%)
Predisposing factors	*Gender*		*Enabling factors*	*Closeness to family and friends*	
	Male	128(39.75%)		Not close	42(13.04%)
	Female	194(60.25%)		Close	138(42.86%)
	*Age*			Very close	142(44.10%)
	60–69	152(47.20%)		*Convenience to community life care center*	
	70–79	131(40.68)		Inconvenient	125(38.82%)
	≥80	39(12.11%)		Convenient	87(27.02%)
	*Education*			Very convenient	110(34.16%)
	Elementary school and below	89(27.64%)		*Convenience to healthcare center*	
	Middle school	100(31.06%)		Inconvenient	82(25.55%)
	High school and above	133(41.30%)		Convenient	100(32.15%)
	*Marital status*			Very convenient	139(43.30%)
	Single	89(27.64%)		*Convenience to recreation center*	
	Not single	233(72.36%)		Inconvenient	99(30.75%)
Need factors	*Chronic disease*			Convenient	92(59.32%)
	Yes	164(50.93%)		Very convenient	131(40.68%)
	No	158(49.07%)		*Proficiency in using smart phone*	
				Strange	222(68.94%)
	*Pension*			Proficient	66(20.50%)
	Yes	309(95.96%)		Very proficient	34(10.56%)
	No	13(4.04%)			
	*Labor income*				
	Yes	83(25.78%)			
	No	239(74.22%)			

Regarding enabling factors, when questioned about their closeness with the community, over half of the respondents expressed that they maintained a frequent contact with others. For spatial linkage measurements, although most old people were satisfied with the convenience to all types of community institutions, 38.82, 25.55, and 30.75% of the respondents reflected that they had some trouble in visiting the nearby community life care center, healthcare center, and recreation center, respectively. For information linkage, 68.94% of the respondents could not operate their smartphones properly.

Moreover, regarding need factors, in general, approximately half of the survey respondents claimed that they had at least one chronic disease. Overall, 96% of the respondents had retirement pension, and 25.78% of the respondents chose to work and had labor income after retirement.

Table 3 presents the characteristics of social psychological factors. Overall, 51.86% of the respondents felt unfair in the process of receiving community care service, and approximately 48% of the respondents felt that they received the same treatments as others in terms of service contents and quality. Regarding vertical fairness, 62.11% of the respondents indicated that their current service benefits did not match their social contribution value before retirement, a sense of being under-served. Approximately 38% of the respondents agreed that they had been valued by the community.

**Table 3:** Descriptive statistics about social psychological factors (*N* = 322).

Variable names		*N* (%)
Social psychological factors	*Be treated equally in community care services*	
	Disagree	167(51.86%)
	Agree	83(25.78%)
	Strongly agree	72(22.36%)
	*Received care services matched with previous social contribution*	
	Disagree	200(62.11%)
	Agree	68(21.12%)
	Strongly agree	54(16.77%)

Table 4 lists the characteristics of dependent variables. Respondents who expressed dissatisfaction with the three different care services accounted for 31.68, 41.61, and 28.88%, respectively. The results showed that people had a relatively higher negative impression about the health care service than about the other two types of services. By contrast, majority of the respondents were satisfied with the community care services they had received.

**Table 4:** Descriptive statistics about dependent variables (*N* = 322).

Variable names		*N* (%)
Community-based service utilization satisfaction	*Life care service satisfaction*	
	Not satisfied	102(31.68%)
	Satisfied	220(68.32%)
	*Healthcare service satisfaction*	
	Not satisfied	134(41.61%)
	Satisfied	188(58.39%)
	*Mental and spiritual service satisfaction*	
	Not satisfied	93(28.88%)
	Satisfied	229(71.12%)

### 3.2. Binary logistic regression

Table 5 presents the results of the binary logistic regression model for satisfaction with the utilization of three service types. We conducted three different regression analyses for the three dependent variables of life care, healthcare, and mental and spiritual service satisfaction. As shown in Table 5, models 1–1, 2–1, and 3–1 all incorporated predisposing characteristics, enabling factors and need factors, and then psychological factors were incorporated in models 1–2, 2–2, and 3–2.

**Table 5:** Results of binary logistic regression for community-based care service utilization satisfaction (*N* = 322).

Variables		Life care service satisfaction		Healthcare service satisfaction		Mental and spiritual service satisfaction	
		Model 1–1 (OR)	Model 1–2 (OR)	Model 2–1 (OR)	Model 2–2 (OR)	Model 3–1 (OR)	Model 3–2 (OR)
Predisposing factors	Age	1.01	1.01	1.02	1.02	0.98	0.97
	Gender (female = 0)	0.59^*^	0.55^**^	0.94	0.81	1.01	0.96
	Marital status (single = 0)	1.99^**^	2.45^***^	1.15^**^	1.41^**^	1.50	1.72
	Education (primary school and below)						
	Middle school	0.59	0.58	0.75^**^	0.89^**^	0.42^***^	0.54^**^
	High school and above	0.70	0.69	0.83^*^	0.84^*^	0.48^*^	0.37^*^
Enabling factors	Closeness to family and friends (not close = 0)						
	Close	1.27	1.19	1.36	1.28	0.62	0.54
	Very close	0.96	0.76	0.58^*^	0.43^*^	0.34^**^	0.25^**^
	Convenience to life care center (inconvenient = 0)						
	Convenient	2.04^***^	2.26^***^				
	Very convenient	2.33^***^	2.26^***^				
	Convenience to healthcare center (inconvenient = 0)						
	Convenient			3.29^***^	3.68^***^		
	Very convenient			3.35^***^	3.59^***^		
	Convenience to mental and spiritual service center (inconvenient = 0)						
	Convenient					7.01^***^	7.75^***^
	Very convenient					4.02^***^	3.79^***^
	Proficiency in using smart phone (strange = 0)						
	Proficient	1.07	0.89	0.97^*^	0.91^*^	2.06^**^	2.07^**^
	Very proficient	0.86	0.70	0.92^*^	0.87^*^	2.99^**^	2.43^**^
Need factors	Having any chronic disease	1.05	1.04	0.64^*^	0.52^**^	0.75	0.67
	Retiring pension (No = 0)	0.91	0.96	0.54	0.56	0.41^*^	0.51^*^
	Labor income (No = 0)	2.24^**^	2.33^*^	3.47^***^	3.62^***^	2.22^***^	2.21^***^
Psychological factors	Treatments matched previous contribution (disagree = 0)						
	Agree		2.03^***^		3.89^**^		1.32
	Strongly agree		2.61^***^		3.77^**^		3.33^**^
	Be treated equally (disagree = 0)						
	Agree		1.38^*^		1.22		2.35^*^
	Strongly agree		1.56		1.06		1.31^*^
	(constant)	1.26	0.75	0.27^***^	0.31^**^	5.43^*^	10.67^*^
	*Pseudo R-square*	0.07	0.15	0.123	0.210	0.14	0.205

β, regression coefficient; ^*^p < 0.05, ^**^p < 0.01, and ^***^p < 0.001.

As shown in Table 5, in models 1–1, 2–1, and 3–1, factors affecting utilization satisfaction levels regarding three service types were different. Regarding predisposing factors, age exhibited no significant relationship with service satisfaction. Gender, marital status, and educational level had different effects on service satisfaction. In life care service, men mostly provided a negative evaluation compared with women (odds ratio [OR] = 0.59). Moreover, people who were married were also more satisfied than those who were single (OR = 1.99). Thus, marital status had a similar relationship with healthcare service (OR = 1.15). Gender exhibited no significant relationship with healthcare service satisfaction, whereas the education level was negatively related; the higher the education level, the more likely the people to be unsatisfied (OR = 0.75; OR = 0.83). The educational level was the only predisposing factor that was significantly related to mental and spiritual care service; people with middle school literacy or high school and above literacy were mostly unsatisfied with the service than those with primary school and below education (OR = 0.42; OR = 0.48).

Regarding enabling factors, old people who were closely linked with family and friends in their communities exhibited lower satisfaction with healthcare services and mental and spiritual services than those who were not (OR = 0.58; OR = 0.34). For spatial linkage, convenience to nearby community service centers such as life care center, healthcare center, and mental and spiritual service center had a positive effect on satisfaction. The satisfaction of respondents who find it convenient or very convenient to visit service centers was approximately three to seven times higher than that of people who do not find it convenient. For information linkage, proficiency in using smart phones negatively affected the evaluation of satisfaction about healthcare service but positively affected the evaluation of satisfaction about the mental and spiritual care service.

Regarding need factors, respondents who had chronic diseases apparently agreed that the healthcare service in the community did not meet their expectation (OR = 0.64). Regarding economic condition, the satisfaction of respondents who received retirement pension was higher with the mental and spiritual service than that of those who did not receive (OR = 0.41). Respondents who had labor income were more satisfied with all three types of service than those without any income.

Models 1–2, 2–2, and 3–2 introduced psychological factors, mainly including horizontal and vertical fairness perceptions in the process of using senior care service in the community. As Table 5 shows, in both sets of models, predisposing, enabling, and need factors, showed consistent relationships between the dependent and independent variables. For the horizontal fairness perception, it apparently affected older adults’ satisfaction in the mental and spiritual service; people who perceived themselves to be receiving fair treatment were more satisfied than those who claimed to receive unfair treatment (OR = 2.35). No significant difference in satisfaction was observed between people who received “equal” and “very equal” services. Moreover, the vertical fairness perception positively and significantly influenced older adults’ satisfaction in all three service types. In general, the satisfaction of older adults who agreed or strongly agreed that the service they received sufficiently matched their previous contribution was approximately three or four times higher than that of those who believed they were not treated fairly compared to their former contribution.

## 4. Discussion

In this study, community-based senior care services included life care service, healthcare service, and mental and spiritual service. Based on an extended Anderson behavior model with social psychological factors, the study revealed that horizontal and vertical fairness perceptions were positively related to the satisfaction of older adults.

The results indicated that the proportion of respondents who were dissatisfied with community healthcare service was higher (41.61%) than that of respondents who were dissatisfied with the other two service types (31.68 and 28.88%). Healthcare service is typically considered to be demanded the most by older adults, but the supply always cannot adequately meet older adults’ needs (Di et al., 2017). Our survey revealed that people with high educational levels expected higher service quality, especially in medical care and recreation activities. Regarding predisposing factors, people with better conditions, such as higher education levels and having a spouse, are more likely to be unsatisfied with community services than others.

Regarding enabling factors, guided by the P-E fit perspective, this study examined that the utilization satisfaction with different types of community-based senior care services varies among personal, spatial, and information linkage. In line with the results of previous studies, the accessibility of care services would allow older adults to use the community care service (17). This is understandable because of physical weakness, older adults depend more on nearby services. In this study, the proficiency in using smart phones negatively affects the satisfaction of using the healthcare service and positively affects the satisfaction of using mental and spiritual service. Because an increasing number of older adults use smartphones, using smartphones was proven beneficial for improving older adults’ health status and psychological condition. However, older adults were likely to have some psychological problems such as depression and emotional disturbances caused by excessive smartphone usage (Wang, 2020). For example, older adults generally tend to be more interested in news and knowledge related to health and illness, the overwhelming information on the internet, much of it being incorrect or inaccurate, will make them more anxious and suspicious. Therefore, with older adults using smartphones more frequently and proficiently, they may become less patient in the process of using the community healthcare service. On a positive note, the use of smartphones provided convenience to older adults, allowing them to participate in community recreational activities.

Regarding needing factors, older adults with chronic diseases rely more on social support and professional medical services. The current community healthcare service supply was still limited in meeting some basic needs and was a type of welfare wherein services were provided for free or at a low price for older adults (30). This study revealed that older adults with chronic diseases were more likely to be unsatisfied with the community healthcare service than others. Because the main research subjects were the urban older adults, they mostly had a retirement pension. Whether or not having a retirement pension only influenced the satisfaction of using the mental and spiritual care service. Different from the retirement pension, additional labor income after retirement significantly and positively affected utilization satisfaction of all three service types. Life care service and healthcare service are linked to old people’s daily life, and recreational activities were only considered as non-necessities. Furthermore, retirement pension was considered a guaranteed item that can only support the old people’s basic daily life, but labor income can help old people seek some other high-quality services.

Regarding psychological factors, our study highlights that the “fairness perception” was positively associated with the satisfaction of using the community-based care service among respondents. As expected, the data revealed that the vertical fairness perception had a more profound influence than the horizontal fairness perception. According to relative deprivation theory, with the increasing age, old people usually tend to reject social help, because their relative deprivation sense decreases with age and so they would not pay any additional attention to chase high-quality life. Consistent with previous findings, older adults were more tolerant with some unfair treatments and were careful in maintaining good relationships with others. Refined and differentiated senior care services are required. The service quality must be improved to ensure that older adults feel being valued. In this study, being treated equally positively affected the utilization satisfaction of the mental and spiritual service. Such services or activities are often arranged in groups with no distinction, and thus, many older adults would easily feel being treated unequally. People tend to have higher demands for entertainment services when their basic living needs are ensured. This is consistent with the results of needing factors that respondents with more economic support tended to demand for more service content and quality beyond basic life care or medical care services. It may suggest that old people tend to care for their subjective perception such as fairness more when they have more money. In the future studies, we can attempt to justify whether older adults with better conditions such as more economic and emotional support would feel more unsatisfied in receiving basic level senior care services.

This study has some limitations. First, owing to the challenges in recruiting respondents, we could not measure aspects such as ethnicity and other potential confounding factors that could affect the service utilization satisfaction of older adults. Additionally, all the data in this study were from the Shaanxi Province, and the results may not be applicable to other regions.

## 5. Conclusion

This study extended the Anderson behavior model from the original three dimensions to four dimensions by including social psychological factors. First, the study reflects different influential factors for three types of community-based senior care service utilization satisfaction. Second, guided by the P-E fit theory, we discussed how personal, spatial, and information linkage worked in the re-adaptation process. Lastly, we testified that in terms of psychological factors, fairness perception was associated with the service utilization satisfaction of older adults. As China’s population is aging rapidly, enhancing the community service quality and improving the service utilization satisfaction among older adults are of great significance.

## Data availability statement

The raw data supporting the conclusions of this article will be made available by the authors, without undue reservation.

## Ethics statement

The studies involving human participants were reviewed and approved by the Ethics Committee of Xi’an Jiaotong University (2016-416; 30 June 2016). The patients/participants provided their written informed consent to participate in this study.

## Author contributions

WM: conceptualization. LL and JW: methodology. HZ: literature review. LL and WM: original draft writing. JW, WM, and LL: revising and editing. All authors have read and agreed to the published version of the manuscript.

## Funding

This study was funded by the Major Projects of Philosophy and Social Science Research of the Ministry of Education (18JZD045) and National Natural Science Fund of China (71764020).

## Conflict of interest

The authors declare that the research was conducted in the absence of any commercial or financial relationships that could be construed as a potential conflict of interest.

## Publisher’s note

All claims expressed in this article are solely those of the authors and do not necessarily represent those of their affiliated organizations, or those of the publisher, the editors and the reviewers. Any product that may be evaluated in this article, or claim that may be made by its manufacturer, is not guaranteed or endorsed by the publisher.
